# Efficient genome editing in rice with miniature Cas12f variants

**DOI:** 10.1007/s42994-024-00168-2

**Published:** 2024-05-28

**Authors:** Zhengyan Ye, Yuanyan Zhang, Shiqi He, Shaokang Li, Longjiong Luo, Yanbiao Zhou, Junjie Tan, Jianmin Wan

**Affiliations:** 1 Sanya Institute of Nanjing Agricultural University, State Key Laboratory of Crop Genetics & Germplasm Enhancement and Utilization, Province and Ministry Co-sponsored Collaborative Innovation Center for Modern Crop Production, Nanjing Agricultural University, Nanjing, 210095 China; 2 Zhongshan Biological Breeding Laboratory, No. 50 Zhongling Street, Nanjing, 210014 China; 3 Key Laboratory of Southern Rice Innovation & Improvement, Ministry of Agriculture and Rural Affairs/Hunan Engineering Laboratory of Disease and Pest Resistant Rice Breeding, Yuan Longping High-Tech Agriculture Co., Ltd, Changsha, 410001 China

**Keywords:** CRISPR/Cas, Cas12, AsCas12f, Rice, Genome editing

## Abstract

Genome editing, particularly using the CRISPR/Cas system, has revolutionized biological research and crop improvement. Despite the widespread use of CRISPR/Cas9, it faces limitations such as PAM sequence requirements and challenges in delivering its large protein into plant cells. The hypercompact Cas12f, derived from *Acidibacillus sulfuroxidans* (AsCas12f), stands out due to its small size of only 422 amino acids and its preference for a T-rich motif, presenting advantageous features over SpCas9. However, its editing efficiency is extremely low in plants. Recent studies have generated two AsCas12f variants, AsCas12f-YHAM and AsCas12f-HKRA, demonstrating higher editing efficiencies in mammalian cells, yet their performance in plants remains unexplored. In this study, through a systematic investigation of genome cleavage activity in rice, we unveiled a substantial enhancement in editing efficiency for both AsCas12f variants, particularly for AsCas12f-HKRA, which achieved an editing efficiency of up to 53%. Furthermore, our analysis revealed that AsCas12f predominantly induces deletion in the target DNA, displaying a unique deletion pattern primarily concentrated at positions 12, 13, 23, and 24, resulting in deletion size mainly of 10 and 11 bp, suggesting significant potential for targeted DNA deletion using AsCas12f. These findings expand the toolbox for efficient genome editing in plants, offering promising prospects for precise genetic modifications in agriculture.

**Supplementary Information:**

The online version contains supplementary material available at 10.1007/s42994-024-00168-2.

Dear Editor,

CRISPR/Cas-mediated genome editing has revolutionized biological research and crop improvement due to its simplicity and cost-effectiveness (Alamillo et al. 2023; Wang and Doudna 2023). This technology enables precise modifications to plant genomes, providing scientists with the capability to improve desired traits, increase crop yield, and enhance resistance against pests and diseases (Hendelman et al. 2021; Huang et al. 2020; Li et al. 2022; Sha et al. 2023; Song et al. 2022; Wang et al. 2019; Zhou et al. 2022).

Despite the extensive use of the CRISPR/Cas9 system, derived from *Streptococcus pyogenes*, it suffers from limitations, including the requirement for PAM NGG in target sites and challenges in delivering its large protein into plant cells, especially with viral vectors or nanoparticles. Advancements have been made to overcome these limitations, including the development of artificial SpCas9 variants with expanded PAM recognition (Tan et al. 2022; Walton et al. 2020) and the exploration of alternative natural Cas systems (Altae-Tran et al. 2023). Notably, recent reports highlight promising CRISPR Type V-F systems, featuring smaller Cas effector proteins (400 to 700 amino acids) with a preference for the TT motif (Bigelyte et al. 2021). AsCas12f, from *Acidibacillus sulfuroxidans*, distinguishes itself with a compact size of only 422 amino acids (Bigelyte et al. 2021), one-third of SpCas9's size. Structural analysis reveals distinctive features, demonstrating an asymmetric homodimeric configuration responsible for dsDNA cleavage activity (Wu et al. 2021), offering insights into unexplored properties and potential applications of Cas12f effectors in genome editing.

While AsCas12f initially demonstrated extremely low activity in plants (Gong et al. 2024), recent enhancements, including deep mutational scanning (DMS) and structural analysis, led to two activity-enhanced variants as well as optimized sgRNA (Hino et al. 2023). Using these AsCas12f variants with optimized sgRNA substantially improved editing efficiency, comparable to SpCas9 and AsCas12a in human cells (Hino et al. 2023). However, their performance in plants has yet to be explored.

For evaluating AsCas12f's potential in rice genome editing, three constructs were designed. The first harbored the wild-type, rice codon-optimized AsCas12f with a single guide RNA (sgRNA) (Wu et al. 2021). The second expressed the engineered variant AsCas12f-YHAM, with four mutations (F48Y/S188H/V232A /E316M), and an optimized sgRNA (sgRNA_ΔS3–5_v7) (Hino et al. 2023). The third expressed another variant, AsCas12f-HKRA. AsCas12f expression, with nuclear localization signals at both termini, was controlled by the *ZmUbi* promoter. Simultaneously, sgRNA expression was driven by *OsU3*, and terminated by the self-cleaving ribozyme HDV and poly T terminator (Fig. 1A; Sequences S1).

**Fig. 1:**
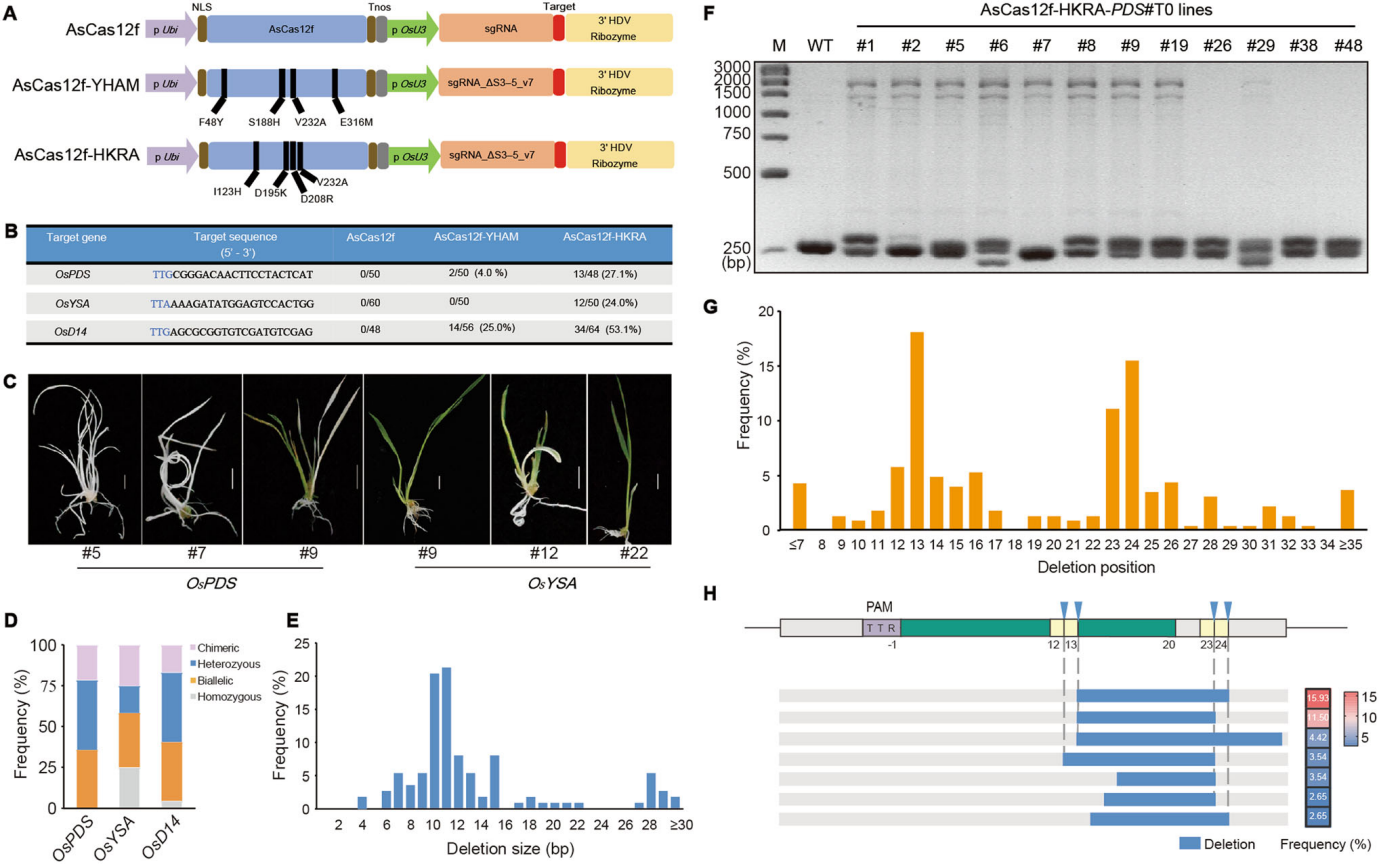
CRISPR/AsCas12f-mediated genome editing in rice. **A** Schematic of AsCas12f and its two activity-enhanced variants, AsCas12f-YHAM and AsCas12f-HKRA, as well as the optimized sgRNA, sgRNA_ΔS3–5_v7. **B** Summary of editing efficiencies of the T0 transgenic rice plants. The target sequences in *OsPDS*, *OsYSA*, and *OsD14* are shown, with PAM sequences highlighted in blue. **C** Albino leaves observed in *OsPDS* and *OsYSA* T0 plants. Scale bar, 0.5 cm. **D** Distribution of homozygous, biallelic, heterozygous, and chimeric mutations in *OsPDS*, *OsYSA*, and *OsD14*. **E** Frequencies of each deletion size in T0 plants. **F** Genotyping of *OsPDS* T0 edited plants using AsCas12f-HKRA variant. **G, H** Deletion pattern of AsCas12f, showing frequencies at each deletion position **(G)** and size **(H)**. Deletion positions were defined as positions from which the deletions originated. Blue arrowheads represented reported cleavage sites (Wu et al. 2021). The frequencies for all deletion sizes are presented in Fig. S5. The heatmaps on the right indicate the percentage of corresponding left deletion size

Three endogenous rice target sites, located within *OsPDS*, *OsYSA*, and *OsD14*, respectively, were selected for evaluation, each containing a TTR motif (R = A or G) at their 5' start (Fig. 1B)*.* Subsequently, nine AsCas12f vectors were transformed into rice calli via Agrobacterium-mediated transformation. After the regeneration of transgenic plants, albino T0 plants were observed in those treated with two AsCas12f variants, but not in those treated with the wild-type AsCas12f (Fig. 1C). Sequence analyses revealed that while no mutations were detected at any of the three target sites in lines treated with wild-type AsCas12f, the lines treated with the two variants exhibited varying degrees of mutagenesis (Fig. 1B).

Compared to the AsCas12f-YHAM variant, with editing frequencies of 4% and 25% in *OsPDS* and *OsD14*, respectively, and no mutations in *OsYSA*, the AsCas12f-HKRA variant displayed superior substrate sequence compatibility and higher nuclease activity. It edited all three target sites, with significantly higher efficiency, showing frequencies of 27%, 24%, and 53% in *OsPDS*, *OsYSA*, and *OsD14*, respectively (Fig. 1B). This represented a remarkable 2.1- and 6.8-fold increase in editing efficiency compared to the AsCas12f-YHAM variant.

Upon analyzing each mutant allele (Fig. S1-S3), it was observed that nearly all mutations (102 out of a total of 113 edited alleles) induced by AsCas12f were deletions, ranging in size from 4 to 54 bp. This differs from the SpCas9-induced mutation pattern, which primarily produces 1 bp insertions (Fig. S4). In *OsPDS* mutants, 42.9% were biallelic, 21.4% chimeric, and 35.7% heterozygous. *OsYSA* mutants exhibited 33.3% heterozygous and 16.7% biallelic mutants, with the remaining mutations at a frequency of 25%. For *OsD14* mutants, 42.9%, 35.7%, 16.7%, and 4.7% represented biallelic, heterozygous, chimeric, and homozygous mutants, respectively (Fig. 1D). Deletion size distribution revealed that deletions predominantly ranged from 7 to 15 bp, constituting 78.8% of all mutations, with 10- (24.3%) and 11-bp deletions (17.8%) being the most frequent (Fig. 1E). This mutation pattern allowed to be efficiently detected using a simple genotype analysis through agarose gel electrophoresis, eliminating the need for costly sequencing (Fig. 1F).

The above results, indicating that nearly all mutations induced by AsCas12f were deletions, suggest its potential for targeted DNA deletion. Analysis of the deletion positions (defined as positions from which the deletions originated) across all 113 generated mutated alleles revealed a concentration at positions 12, 13, 23, and 24 relative to the PAM sequence, particularly the latter three positions (Fig. 1G). This concentration resulted in the predominant generation of 10- and 11-bp DNA deletions (Fig. 1H; Fig. S5). It is noteworthy that these positions were previously reported as cleavage sites as well (Wu et al. 2021).

In summary, our study demonstrated, for the first time, the effectiveness of the miniature AsCas12f variants in rice, particularly the variant AsCas12f-HKRA, thus expanding the toolkit for efficient genome editing in plants. Moreover, our findings revealed a unique deletion pattern of AsCas12f, indicating its significant potential for targeted DNA deletion applications.

## Materials and methods

### Vector construction

PCR was conducted using Phanta Max Super-Fidelity DNA Polymerase (Vazyme Biotech) following the manufacturer’s instructions. To generate AsCas12f as well as its variants, sequences were synthesized (Tsingke Biotech) and cloned into the BamHI-digested vectors pYLCRISPR/Cas9Pubi-B (Ma et al. 2015b). In addition, sgRNA scaffold and its optimized version sgRNA_ΔS3–5_v7 were synthesized and ligated into BsaI-digested vectors containing AsCas12f or its variants, resulting in the generation of AsCas12f-sgRNA, AsCas12f-YHAM + sgRNA_ΔS3–5_v7, and AsCas12f -HKRA + sgRNA_ ΔS3–5_v7. To target specific genome sites in *OsPDS*, *OsYSA*, and *OsD14*, a spacer sequence was identified in the target region and introduced into above AsCas12f vectors using Golden Gate ligation. All primers are listed in Table S1. All DNA sequences are listed in Sequences S1.

### Rice transformation

The constructs were introduced into *Agrobacterium tumefaciens* strain EHA105 and subsequently transformed into embryogenic calli induced from rice variety Ningjing 7 (*Oryza sativa* L. ssp. japonica) mature seeds. Rice transformation, tissue culture, and plantlet growth were carried out following established procedures (*Oryza sativa* L. ssp. japonica) using established procedures (Duan et al. 2012). Three days post-transformation, the calli were transferred to N6 medium supplemented with 2,4-dichlorophenoxyacetic acid, casamino acids, gelrite, sucrose, carbenicillin, and hygromycin for a duration of 21 days. The resulting resistant calli were selected and further transferred to regeneration media for 14 days. After 2 weeks of rooting, the regenerated plantlets were subsequently transplanted into a greenhouse under standard growth conditions (14 h of light alternating with 10 h of darkness at 28 °C).

### Genotyping of targeted mutations

The genomic DNA from rice plant leaves was isolated using the CTAB method. The genomic region containing the target site was amplified in the respective transgenic plants using site-specific primers (Table S1). The resulting products were then subjected to agarose gel electrophoresis or analyzed by Sanger sequencing. For mutation analysis, the sequencing chromatograms were analyzed using DSDecode (Liu et al. 2015; Ma et al. 2015a). The primer sets used for PCR and sequencing are listed in Table S1.

### Supplementary Information

Below is the link to the electronic supplementary material.

Supplementary file1 (PDF 1451 KB)

## Data Availability

Datasets generated during the current study are available from the corresponding author upon reasonable request.
