# Supplementary material for: Efficient genome editing in rice with miniature Cas12f variants
Source: aBIOTECH. 2024 May 28;5(2):184–8. doi: 10.1007/s42994-024-00168-2 (PMC11224166; doi:10.1007/s42994-024-00168-2)
Supplement: Supplementary file 1 — Supplementary file1 (PDF 1451 KB) [file 42994_2024_168_MOESM1_ESM.pdf]

Efficient genome editing in rice with miniature Cas12f variants

Zhengyan Ye[1, 2, a], Yuanyan Zhang[1, 2, a], Shiqi He[1, 2, a], Shaokang Li[1, 2], Longjiong Luo[1, 2], Yanbiao Zhou[3], Junjie Tan[1, 2, *], Jianmin Wan[1, 2, *]

[1]Sanya Institute of Nanjing Agricultural University, State Key Laboratory of Crop Genetics & Germplasm Enhancement and Utilization, Province and Ministry Co-sponsored Collaborative Innovation Center for Modern Crop Production, Nanjing Agricultural University, Nanjing, 210095, China

[2]Zhongshan Biological Breeding Laboratory, No. 50 Zhongling Street, Nanjing, 210014, China

[3]Key Laboratory of Southern Rice Innovation & Improvement, Ministry of Agriculture and Rural Affairs/Hunan Engineering Laboratory of Disease and Pest Resistant Rice Breeding, Yuan Longping High-Tech Agriculture Co., Ltd, Changsha, 410001, China

[a]These authors contributed equally to this article.

[*]Correspondence (e-mail: tanjunjie@njau.edu.cn (JT); wanjm@njau.edu.cn (JW))

Supplementary Information

Table of contents

Fig. S1 Mutations induced by AsCas12f at the *OsPDS* target site 3

Fig. S2 Mutations induced by AsCas12f at the *OsYSA* target site... 4

Fig. S3 Mutations induced by AsCas12f at the *OsD14* target site 5

Fig. S4 Frequency of SpCas9-induced mutations at the *OsYSA* target site 7

Fig. S5 Schematic representation of the deletion pattern for a total of 113 AsCas12f-edited alleles.. 8

Table S1 Primer sequences used in this study 10

Sequence S1 DNA sequences of AsCas12f, its two variants, and the sgRNA scaffold used in this study with the mutation sites highlighted in red 11

PDS#reference	CTGGAACCAGCCAAGCAAGATCTTTTGCGGGACAACTTCCTACTCATAGGTGCTTCGCAAGTAGCAGCAT		
HKRA- PDS#1	CTGGAACCAGCCAAGCAAGATCTTTTGCGGGACAACTTCC-----------GCTTCGCAAGTAGCAGCAT	-11	Biallelic
	CTGGAACCAGCCAAGCAAGATCTTTTGCGGGACAACTTCCTA-----tgagGCTTCGCAAGTAGCAGCAT	-9/+4	
HKRA- PDS#2	CTGGAACCAGCCAAGCAAGATCTTTTGCGGGACAACTTCCTAC-------TGCTTCGCAAGTAGCAGCAT	-7	Biallelic
	CTGGAACCAGCCAAGCAAGATCTTTTGCGGGACAACTTCCTA--------TGCTTCGCAAGTAGCAGCAT	-8	
HKRA- PDS#5	CTGGAACCAGCCAAGCAAGATCTTTTGCGGGACAACTTCCTAC-------TGCTTCGCAAGTAGCAGCAT	-7	Biallelic
	CTGGAACCAGCCAAGCAAGATCTTTTGCGGGACAACTTCC----------TGCTTCGCAAGTAGCAGCAT	-10	
HKRA- PDS#6	CTGGAACCAGCCAAGCAAGATCTT-----------------------------TTCGCAAGTAGCAGCAT	-29	Biallelic
	CTGGAACCAGCCAAGCAAGATCTTTTGCGGGACAACTTCCTACTCAT----GCTTCGCAAGTAGCAGCAT	-4	
HKRA- PDS#7	CTGGAACCAGCCAAGCAAGATCTTTTGCGGGACAACTTCCTA----------CTTCGCAAGTAGCAGCAT	-10	Biallelic
	CTGGAACCAGCCAAGCAAGATCTTTTGCGGGACAACTTCC-----------GCTTCGCAAGTAGCAGCAT	-11	
HKRA- PDS#8	CTGGAACCAGCCAAGCAAGATCTTTTGCGGGACAACTTCCTA----------CTTCGCAAGTAGCAGCAT	-10	Biallelic
	CTGGAACCAGCCAAGCAAGATCTTTTGCGGGACAACTTCCTAC-----ctTGCTTCGCAAGTAGCAGCAT	-7/+2	
HKRA- PDS#9	CTGGAACCAGCCAAGCAAGATCTTTTGCGGGACAACTTC------------GCTTCGCAAGTAGCAGCAT	-12	Hetero
HKRA- PDS#11	CTGGAACCAGCCAAGCAAGATCTTTTGCGGGACAACTTCC----------TGCTTCGCAAGTAGCAGCAT	-10	Hetero
HKRA- PDS#19	CTGGAACCAGCCAAGCAAGATCTTTTGCGGGACAACTTCC----------TGCTTCGCAAGTAGCAGCAT	-10	Hetero
HKRA- PDS#26	CTGGAACCAGCCAAGCAAGATCTTTTGCGGGACAACTTCCTA--------TGCTTCGCAAGTAGCAGCAT	-8	Hetero
HKRA- PDS#29	CTGGAACCAGCCAAGCAAGATCTTTTGCGGGACAACTTCCT----------------------------T	-28	Biallelic
	CTGGAACCAGCCAAGCAAGATCTTTTGCGGGACAAC----------------------------CAGCAT	-28	
HKRA- PDS#38	CTGGAACCAGCCAAGCAAGATCTTTTGCGGGACAACTTCCTAC--------GCTTCGCAAGTAGCAGCAT	-8	Hetero
HKRA- PDS#48	CTGGAACCAGCCAAGCAAGATCTTTTGCGGGACAACTTCC----------TGCTTCGCAAGTAGCAGCAT	-10	Chimeric
	CTGGAACCAGCCAAGCAAGAT----------ACAACTTCCTACTCATAGGTGCTTCGCAAGTAGCAGCAT	-10	
YHAM- PDS#6	CTGGAACCAGCCAAGCAAGATCTTTTGCGGGACAACTTC-------------CTTCGCAAGTAGCAGCAT	-13	Chimeric
	CTGGAACCAGCCAAGCAAGATCTTTTGCGG-------------TCATAGGTGCTTCGCAAGTAGCAGCAT	-13	

Fig. S1 Mutations induced by AsCas12f at the *OsPDS* target site. The deleted nucleotides were indicated by '-'. The TTR PAM and 20-bp spacer sequence were highlighted by red and blue, respectively. The red lowercase sequences represent insertions. Genotype information is provided on the right. '-n' denotes nucleotide deletion, and '-n/+n' denotes simultaneous nucleotide deletion/insertion. For heterozygous and chimeric lines, only mutated allele sequences were displayed.

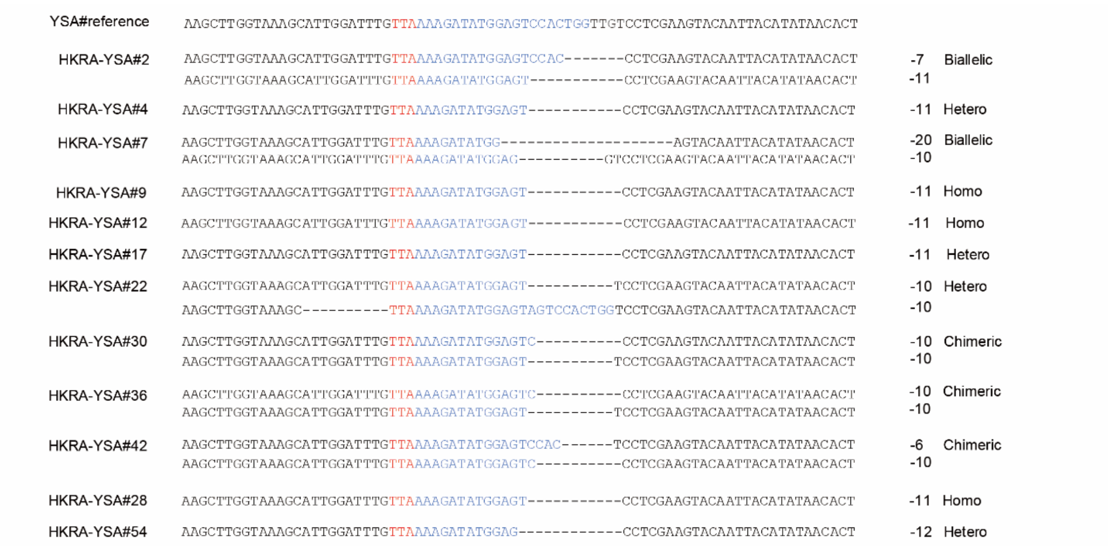

Fig. S2 Mutations induced by AsCas12f at the *OsYSA* target site. The deleted nucleotides were indicated by '-'. The TTR PAM and 20-bp spacer sequence were highlighted by red and blue, respectively. Genotype information is provided on the right. '-n' denotes nucleotide deletion.

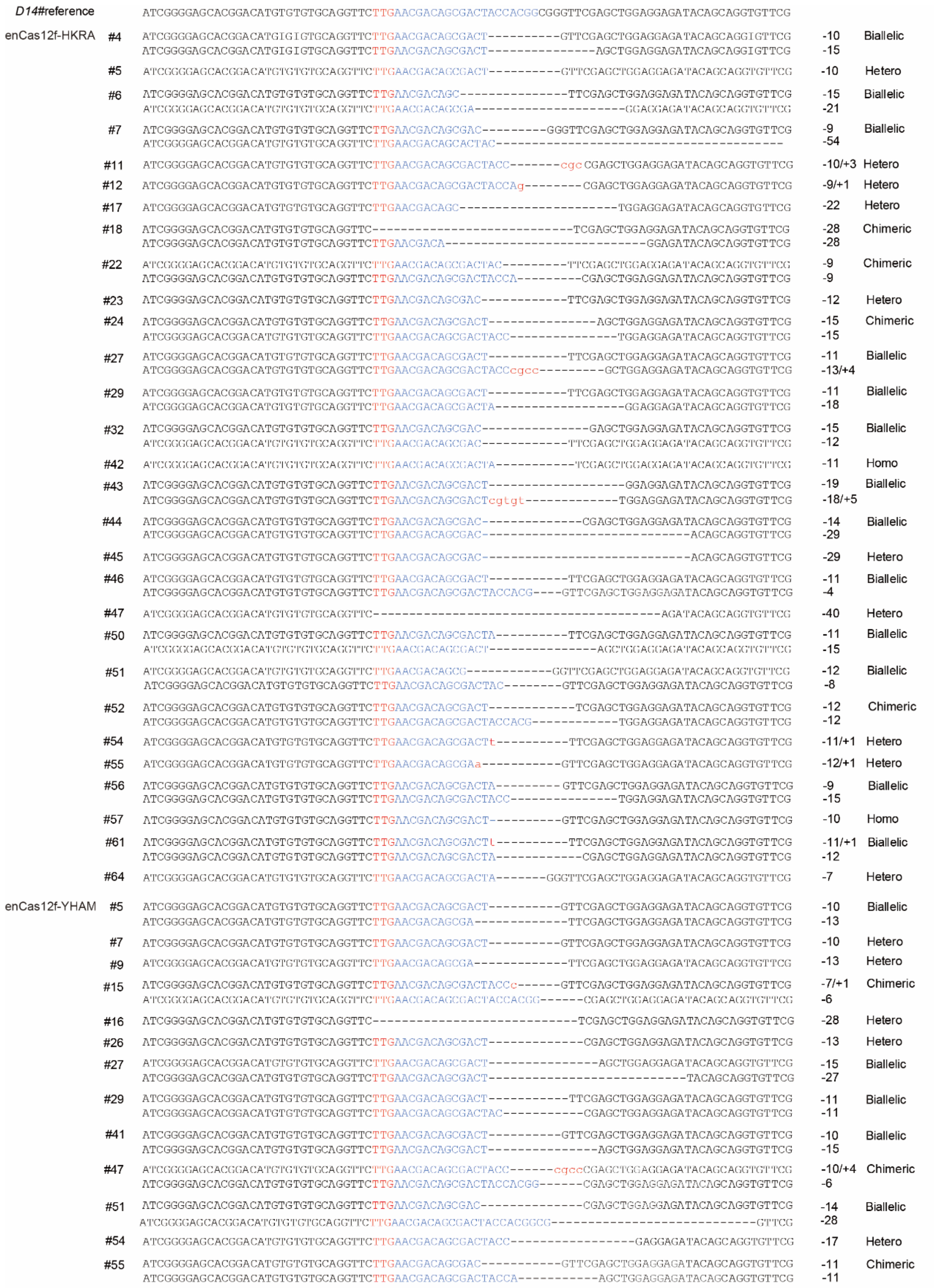

Fig. S3 Mutations induced by AsCas12f at the *OsD14* target site. The deleted nucleotides were indicated by '-'. The TTR PAM and 20-bp spacer sequence were highlighted by red and blue, respectively. The red lowercase sequences represent

insertions. Genotype information is provided on the right. '-n' denotes nucleotide deletion, and '-n/+n' denotes simultaneous nucleotide deletion/insertion.

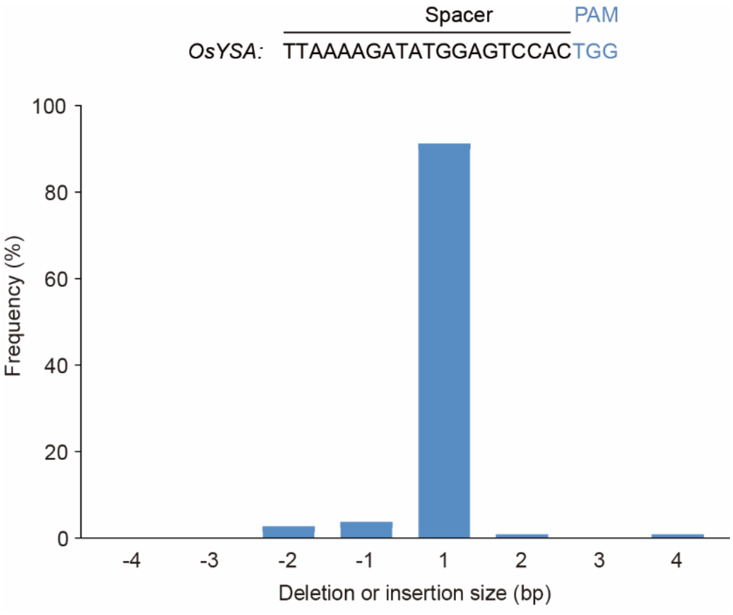

Fig. S4 Frequency of SpCas9-induced mutations at the *OsYSA* target site. A construct expressing SpCas9 and sgRNA, targeting the rice *OsYSA* gene, was transformed into rice calli, and a total of 52 T0 lines were edited, resulting in 103 mutant alleles, which were analyzed.

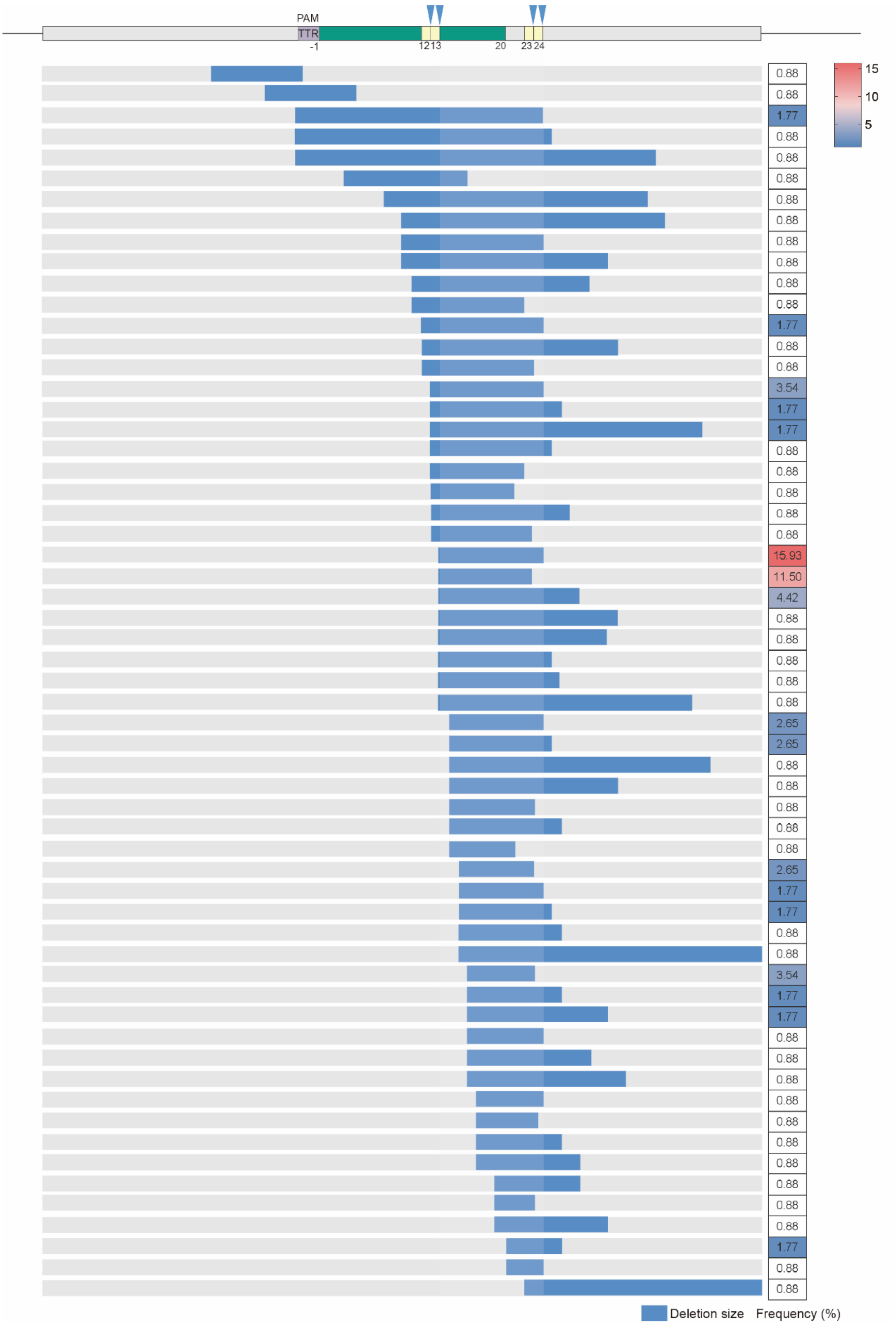

Fig. S5 Schematic representation of the deletion pattern for a total of 113 AsCas12f-edited alleles. The TTR PAM and spacer sequences were highlighted in purple and green. The yellow box represents the key nucleotides at which the break sites were located, with

the numbers below indicating their position relative to the PAM. Blue arrowheads represented reported cleavage sites. A series of blue boxes listed below represents deletions of various sizes. The heatmaps on the right indicate the percentage of corresponding left deletion size.

Table S1 Primer sequences used in this study.

Primers Name	Primer Sequence (5’-3’)	Purpose
oJT498-pJTP21-1F	AGGTAGCGAAGGATCCGCTC	Construct of Cas12f and its variants
oJT499-pJTP21-1R	GTTGAGGGCGAACCTTGTCTCCTGCTGGAGCTGCC	
oJT500-pJTP21-2F	AGGTTCGCCCTCAACAAGG	
oJT501-pJTP21-2R	ACGATCGGGAGGATCCTAC	
oJT472-pJTP20-1F	GCGCCGTAGTGCTCGAAGGAATCTTTAAACATACGAAC	Introduction of sgRNA scaffold and 3' HDV Ribozyme
oJT473-pJTP20-1R	TGCCACGGATCATCTGCAC	
oJT474-pJTP20-2F	AGATGATCCGTGGCAGGGATTCGTCGGTTCAGCGACG	
oJT475-pJTP20-2R	GCGCCAATGATACCGAGACATAAAAAACAAAAAAAGTCCCATTCGCCATGCCG	
sgRNA-*OsPDS*-F	GGCCATGAGTAGGAAGTTGTCCCG	Targeting endogenous *OsPDS* gene
sgRNA-*OsPDS*-R	GAACCGGGACAACTTCCTACTCAT	
sgRNA-*OsYSA*-F	GGCCCCAGTGGACTCCATATCTTT	Targeting endogenous *OsYSA* gene
sgRNA-*OsYSA*-R	GAACAAAGATATGGAGTCCACTGG	
sgRNA-*OsD14*-F	GGCCCCGTGGTAGTCGCTGTCGTT	Targeting endogenous *OsD14* gene
sgRNA-*OsD14*-R	GAACAACGACAGCGACTACCACGG	
sgRNA-Cas9-*OsYSA*-F	GGCGTTAAAAGATATGGAGTCCAC	Targeting endogenous *OsYSA* gene
sgRNA-Cas9-*OsYSA*-R	AAACGTGGACTCCATATCTTTTAA	
OsPDS-F	TTGTGCCATGCTTGATCTGTT	For PCR amplification and sequencing of of *OsPDS*
OsPDS-R	AGGTCTTGGAAAGTCCTGGC	
OsYSA-F	TCCGCATACCTCTGAGTACC	For PCR amplification and sequencing of *OsYSA*
OsYSA-R	GGGGTTGTTTGCCTGACATTA	
OsD14-F	AGAGTCAAGACCATCTCCCAT	For PCR amplification and sequencing of *OsD14*
OsD14-R	GCCCACGCCGAGTAGTT	
PDS-ch-F	TGTTTTTGCAGACGCTCTTG	For genotyping
PDS-ch-R	TCCGACGGAATCTTTTTCCT	

Sequence S1 DNA sequences of AsCas12f, its two variants, and the sgRNA scaffold used in this study with the mutation sites highlighted in red.

AsCas12f

ATGATCAAGGTGTACAGGTACGAGATCGTGAAGCCGCTCGACCTCGACTGGAAGGA
GTTCGGCACCATCCTCAGGCAGCTCCAGCAGGAGACAAGGTTCGCCCTCAACAAGG
CCACCCAGCTCGCCTGGGAGTGGATGGGCTTCTCCTCCGACTACAAGGACAACCAC
GGCGAGTACCCGAAGTCCAAGGACATCCTCGGCTACACCAACGTGCACGGCTACGC
CTACCACACCATCAAGACCAAGGCCTACAGGCTCAACTCCGGCAACCTCTCCCAGA
CCATCAAGAGGGCCACCGACAGGTTCAAGGCCTACCAGAAGGAGATCCTCAGGGG
CGACATGTCCATCCCGTCCTACAAGAGGGACATCCCGCTCGACCTCATCAAGGAGA
ACATCTCCGTGAACAGGATGAACCACGGCGACTACATCGCCTCCCTCTCCCTCCTCT
CCAACCCGGCCAAGCAGGAGATGAACGTGAAGAGGAAGATCTCCGTGATCATCATC
GTGAGGGGCGCCGGCAAGACCATCATGGACAGGATCCTCTCCGGCGAGTACCAGG
TGTCCGCCTCCCAGATCATCCACGACGACAGGAAGAACAAGTGGTACCTCAACATCT
CCTACGACTTCGAGCCGCAGACCAGGGTGCTCGACCTCAACAAGATCATGGGCATC
GACCTCGGCGTGGCCGTGGCCGTGTACATGGCCTTCCAGCACACCCCGGCCAGGT
ACAAGCTCGAGGGCGGCGAGATCGAGAACTTCAGGAGGCAGGTGGAGTCCAGGAG
GATCTCCATGCTCAGGCAGGGCAAGTACGCCGGCGGCGCCAGGGGCGGCCACGG
CAGGGACAAGAGGATCAAGCCGATCGAGCAGCTCAGGGACAAGATCGCCAACTTCA
GGGACACCACCAACCACAGGTACTCCAGGTACATCGTGGACATGGCCATCAAGGAG
GGCTGCGGCACCATCCAGATGGAGGACCTCACCAACATCAGGGACATCGGCTCCAG
GTTCCTCCAGAACTGGACCTACTACGACCTCCAGCAGAAGATCATCTACAAGGCCGA
GGAGGCCGGCATCAAGGTGATCAAGATCGACCCGCAGTACACCTCCCAGAGGTGCT
CCGAGTGCGGCAACATCGACTCCGGCAACAGGATCGGCCAGGCCATCTTCAAGTGC
AGGGCCTGCGGCTACGAGGCCAACGCCGACTACAACGCCGCCAGGAACATCGCCA
TCCCGAACATCGACAAGATCATCGCCGAGTCCATCAAG

AsCas12f-YHAM

ATGATCAAGGTGTACAGGTACGAGATCGTGAAGCCGCTCGACCTCGACTGGAAGGA
GTTCGGCACCATCCTCAGGCAGCTCCAGCAGGAGACAAGGTTCGCCCTCAACAAGG
CCACCCAGCTCGCCTGGGAGTGGATGGGCTACTCCTCCGACTACAAGGACAACCAC
GGCGAGTACCCGAAGTCCAAGGACATCCTCGGCTACACCAACGTGCACGGCTACGC
CTACCACACCATCAAGACCAAGGCCTACAGGCTCAACTCCGGCAACCTCTCCCAGA
CCATCAAGAGGGCCACCGACAGGTTCAAGGCCTACCAGAAGGAGATCCTCAGGGG
CGACATGTCCATCCCGTCCTACAAGAGGGACATCCCGCTCGACCTCATCAAGGAGA
ACATCTCCGTGAACAGGATGAACCACGGCGACTACATCGCCTCCCTCTCCCTCCTCT
CCAACCCGGCCAAGCAGGAGATGAACGTGAAGAGGAAGATCTCCGTGATCATCATC
GTGAGGGGCGCCGGCAAGACCATCATGGACAGGATCCTCTCCGGCGAGTACCAGG
TGCACGCCTCCCAGATCATCCACGACGACAGGAAGAACAAGTGGTACCTCAACATCT
CCTACGACTTCGAGCCGCAGACCAGGGTGCTCGACCTCAACAAGATCATGGGCATC
GACCTCGGCGTGGCCGTGGCCGCGTACATGGCCTTCCAGCACACCCCGGCCAGGT

ACAAGCTCGAGGGCGGCGAGATCGAGAACTTCAGGAGGCAGGTGGAGTCCAGGAG
GATCTCCATGCTCAGGCAGGGCAAGTACGCCGGCGGCGCCAGGGGCGGCCACGG
CAGGGACAAGAGGATCAAGCCGATCGAGCAGCTCAGGGACAAGATCGCCAACTTCA
GGGACACCACCAACCACAGGTACTCCAGGTACATCGTGGACATGGCCATCAAGATG
GGCTGCGGCACCATCCAGATGGAGGACCTCACCAACATCAGGGACATCGGCTCCAG
GTTCCTCCAGAACTGGACCTACTACGACCTCCAGCAGAAGATCATCTACAAGGCCGA
GGAGGCCGGCATCAAGGTGATCAAGATCGACCCGCAGTACACCTCCCAGAGGTGCT
CCGAGTGCGGCAACATCGACTCCGGCAACAGGATCGGCCAGGCCATCTTCAAGTGC
AGGGCCTGCGGCTACGAGGCCAACGCCGACTACAACGCCGCCAGGAACATCGCCA
TCCCGAACATCGACAAGATCATCGCCGAGTCCATCAAG

AsCas12f-HKRA

ATGATCAAGGTGTACAGGTACGAGATCGTGAAGCCGCTCGACCTCGACTGGAAGGA
GTTCGGCACCATCCTCAGGCAGCTCCAGCAGGAGACAAGGTTCGCCCTCAACAAGG
CCACCCAGCTCGCCTGGGAGTGGATGGGCTTCTCCTCCGACTACAAGGACAACCAC
GGCGAGTACCCGAAGTCCAAGGACATCCTCGGCTACACCAACGTGCACGGCTACGC
CTACCACACCATCAAGACCAAGGCCTACAGGCTCAACTCCGGCAACCTCTCCCAGA
CCATCAAGAGGGCCACCGACAGGTTCAAGGCCTACCAGAAGGAGATCCTCAGGGG
CGACATGTCCATCCCGTCCTACAAGAGGGACCACCCGCTCGACCTCATCAAGGAGA
ACATCTCCGTGAACAGGATGAACCACGGCGACTACATCGCCTCCCTCTCCCTCCTCT
CCAACCCGGCCAAGCAGGAGATGAACGTGAAGAGGAAGATCTCCGTGATCATCATC
GTGAGGGGCGCCGGCAAGACCATCATGGACAGGATCCTCTCCGGCGAGTACCAGG
TGTCCGCCTCCCAGATCATCCACAAGGACAGGAAGAACAAGTGGTACCTCAACATCT
CCTACAGGTTCGAGCCGCAGACCAGGGTGCTCGACCTCAACAAGATCATGGGCATC
GACCTCGGCGTGGCCGTGGCCGCGTACATGGCCTTCCAGCACACCCCGGCCAGGT
ACAAGCTCGAGGGCGGCGAGATCGAGAACTTCAGGAGGCAGGTGGAGTCCAGGAG
GATCTCCATGCTCAGGCAGGGCAAGTACGCCGGCGGCGCCAGGGGCGGCCACGG
CAGGGACAAGAGGATCAAGCCGATCGAGCAGCTCAGGGACAAGATCGCCAACTTCA
GGGACACCACCAACCACAGGTACTCCAGGTACATCGTGGACATGGCCATCAAGGAG
GGCTGCGGCACCATCCAGATGGAGGACCTCACCAACATCAGGGACATCGGCTCCAG
GTTCCTCCAGAACTGGACCTACTACGACCTCCAGCAGAAGATCATCTACAAGGCCGA
GGAGGCCGGCATCAAGGTGATCAAGATCGACCCGCAGTACACCTCCCAGAGGTGCT
CCGAGTGCGGCAACATCGACTCCGGCAACAGGATCGGCCAGGCCATCTTCAAGTGC
AGGGCCTGCGGCTACGAGGCCAACGCCGACTACAACGCCGCCAGGAACATCGCCA
TCCCGAACATCGACAAGATCATCGCCGAGTCCATCAAG

sgRNA_△S3-5_v7

TCGTCGGTTCAGCGACGATAAGCCGAGAAGTGCCAATAAAACTGTTAAGTGGTTTGG

TAACGCTCGGTAAGGTCCGAAAGGAGAACCACTGAAC
